# BRUNNER GLAND ADENOMA MASQUERADING AS DUODENAL GASTROINTESTINAL STROMAL TUMOR WITH INTUSSUSCEPTION: CASE REPORT

**DOI:** 10.1590/0102-6720201700010020

**Published:** 2017

**Authors:** Gunjan DESAI, Kamal YADAV, Prasad PANDE, Priyanka SALI, Chandralekha TAMPI, Prasad WAGLE

**Affiliations:** 1 Department of Gastrointestinal Surgery; 2 Pathology, Lilavati Hospital and Research Center, Bandra Reclamation, Bandra West, Mumbai 400050, Maharashtra, India.

**Keywords:** Duodenal neoplasms, Hamartoma, Intussusception

## INTRODUCTION

Brunner gland adenoma (Brunerroma or hamartoma) is a rare, benign lesion of the Brunner's glands, accounting for 10.6% of benign duodenal tumors^10^. It is predominantly seen in the 5^th^ to 6^th^ decades and with no gender predilection^13^. It is often an incidental finding during esophagogastroduodenoscopy or imaging studies. In symptomatic patients, clinical manifestations include gastrointestinal bleeding, duodenal obstruction, abdominal pain, ampullary obstruction, or intussusception^8^ ^,^ ^9^. Given their potential to be mistaken as cancer, it is important to consider it in the differential diagnosis of duodenal masses^14^. As there have been reports of focal cellular atypia and adenocarcinoma within the lesion, resection, whether endoscopic or surgical is recommended for suspected Brunneromas^3^. 

We report here a case of Brunneroma, which presented as gastrointestinal stromal tumor (GIST) with intussusception on radiological and endoscopic studies and brief review of literature.

## CASE REPORT

**FIGURE 1:**
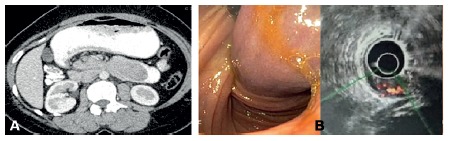
A) CT image shows an intraluminal polypoidal lesion in second part of duodenum with mild duodenal wall thickening; B) endoscopy with EUS shows a large mixed echogenic submucosal lesion of size 4.0x2.8 cm with a 2 cm thick, long pedicle and a feeding artery on color Doppler leading to duodenal intussusception

A 33 year old female presented with vague epigastric discomfort, weakness and breathlessness on exertion, 3-4 episodes of melena and intermittent non-bilious vomiting episodes since 45 days. Her general physical examination revealed pallor. Abdomen showed mild epigastric fullness. Hemoglobin was 6.7 g/dl. Leucocyte count, liver and kidney function tests were within normal limits. Ultrasonography of the abdomen and pelvis revealed a 4.4x3.0x2.7 cm well defined isoechoeic solid mass within bowel lumen possibly in the second part of the duodenum. A contrasted tomography of the abdomen and pelvis (Figure 1A) revealed an intraluminal polypoidal lesion in the second part of duodenum with mild thickening of duodenal wall with few subcentimetric lymph nodes.

Upper gastrointestinal endoscopy with endoscopic ultrasound (EUS, Figure 1B) showed a mixed echogenic submucosal lesion measuring 4.0x2.8 cm with a 2 cm thick, long pedicle and a feeding artery on color Doppler leading to duodenal intussusception and obstruction extending beyond the second part of duodenum. It was diagnosed as GIST based on endoscopic and EUS findings. EUS fine needle cytology or biopsy was not deemed necessary. The patient was planned for pancreas preserving duodenectomy with a pancreaticoduodenectomy if ampulla was involved. The patient was transfused two packed cells preoperatively. 

**FIGURE 2:**
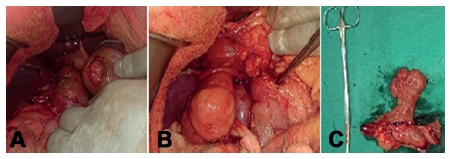
Intraoperative pictures showing: A) duodenotomy at the junction of first and second part of duodenum reveals the lesion, B) lesion exteriorized from duodenum with stalk based in duodenum (instrument is at ampulla); C) surgical specimen

**FIGURE 3:**
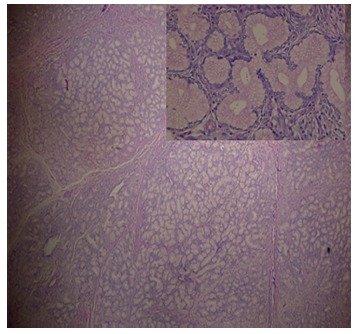
Histopathology: a lobular proliferation of Brunner's glands, accompanied by few ducts and scattered stromal elements, ulcerated surface duodenal epithelium and granulation tissue. Enlarged view shows Brunner's glands

Intraoperatively (Figure 2), an approximately 4x3 cm lesion in duodenal bulb and second part of duodenum with a 5 cm long stalk was arising from the posterior wall of first part of duodenum with ampulla at 3 cm distal to the root. Intraoperative frozen section confirmed the lesion as brunneroma. A supra-ampullary pyloroduodenectomy with Roux-en-y gastrojejunostomy was done. The specimen showed a lobulated, polypoid mass of 4×3×3 cm projecting into the duodenum. The stalk was 5.5x2.0 cm. The tumor was completely enveloped by duodenal mucosa. The surface was smooth and consistency was firm. Histopathology (Figure 3) revealed a lobular proliferation of benign Brunner's glands, accompanied by few ducts and scattered stromal elements, ulcerated surface duodenal epithelium and granulation tissue at places. Few foam cell aggregates were seen and center of the stalk was fibrovascular with normal overlying epithelium. No dysplasia or malignancy was seen. The patient recovered uneventfully and is symptom free at follow up.

## DISCUSSION

Brunner glands are branched acinotubular glands located mainly in the duodenal bulb, proximal duodenum, and, rarely in the distal duodenum and proximal jejunum. They secrete an alkaline fluid which protects the duodenal epithelium from acid chyme of the stomach^12^. Brunner in 1688 described it as "pancreas secundarium". In 1846, Middeldorp identified these submucosal duodenal glands as a separate entity. Salvioli reported the first Brunneroma in 1876^4^. A series of 27 patients with Brunner gland hamartoma found 70% in the duodenal bulb, 26% in the second portion and 4% in third portion of duodenum^9^. In this case it was present in duodenal second part with the stalk in first part. 

How Brunner's gland hamartoma grows is unclear. Repeated mucosal damages activate mucosal repair, facilitating proliferation accompanied by surface gastric foveolar metaplasia. Mechanical stimuli, *Helicobacter pylori* infection, and hyper acidic environment in duodenum have also been suggested though none proven so far^1^. 

It is often an incidental finding during esophagogastroduodenoscopy or imaging studies^9^. The most common presentations in symptomatic patients are gastrointestinal bleeding (37%) and obstructive symptoms (37%)^4^. In a review of 27 cases, Levine et al. found that the majority of patients had melena, iron deficiency anemia with evidence of chronic bleeding^9^. Other symptoms include abdominal pain, pancreatitis, jaundice, or intussusception^9^ ^,^ ^8^. 

The lesion must be differentiated from adenoma, GIST, lipoma, neurogenic tumor, aberrant pancreatic tissue and cystic dystrophy of the duodenal wall^14^. Differential diagnosis in this case was with GIST, as revealed by endoscopic findings. The intense vascularity and the long stalk was the reason to label the lesion as GIST.

Endoscopically, Brunner's gland hyperplasia appears as submucosal nodules in the first or second portion of the duodenum. Endoscopic ultrasound shows a heterogeneous, hypoechoic mass in the submucosal layer^11^. On barium examination, Brunner's gland hyperplasia appears as one or more small nodules in the proximal duodenum with rarely a cobblestone or Swiss cheese pattern. The differential diagnosis include familial adenomatous polyposis, Peutz-Jeghers syndrome, nodular lymphoid hyperplasia, heterotopia, carcinoid tumors, and metastasis^5^. On CT, Brunner's gland hamartoma has been described as having variable echogenicity. Multiple cysts within the hamartoma may produce a more heterogeneous pattern. The homogeneous pattern correlates with histologic findings of glandular proliferation. Conversely, the heterogeneous enhancement pattern correlates with finding of fat and smooth muscle proliferation in addition to glandular proliferation. These are enhancing masses^2^ ^,^ ^11^. 

The abnormal proliferation of Brunner glands are classified as type 1, diffuse nodular hyperplasia with multiple sessile projections throughout the duodenum; type 2, circumscribed nodular hyperplasia with sessile projections limited to the duodenal bulb; and type 3, glandular adenoma with polypoid tumor-like projections. Hyperplasia of Brunner glands greater than 1 cm is a Brunner gland adenoma^15^.

Three to four case reports of the approximately 150 cases reported in literature have shown cellular atypia and malignany. However, as far as treatment is considered, endoscopic resection is also an accepted treatment choice owing to a negligible risk^3^. 

Treatment options can include endoscopic or surgical resection. The benign nature, and the lack of significant symptoms makes endoscopic management the preferred initial treatment. However, if endoscopic interventions fail or if there is a diagnostic dilemma, or for large lesions, or those in whom a malignancy is suspected surgical resection may be necessary^7^. Outcomes are excellent with no reported recurrences after complete resection so far. Occasional reports of pancreaticoduodenectomy have also been reported in view of diagnostic surprise or suspicion of malignancy^6^. Diagnostic surprise, large size with a vascular stalk and posterior duodenal wall involvement in this case promoted a supra-ampullary pyloroduodenectomy.
